# Efficacy of ursodeoxycholic acid for bile reflux after distal gastrectomy in patients with gastric cancer: a secondary analysis of the PEGASUS-D randomized clinical trial

**DOI:** 10.1097/JS9.0000000000002127

**Published:** 2024-10-18

**Authors:** Dong Kee Jang, Young Suk Park, Moon-Won Yoo, Sun-Hwi Hwang, Seong-Yeob Ryu, Oh Kyoung Kwon, Hoon Hur, Hong Man Yoon, Bang Wool Eom, Hye Seong Ahn, Taeil Son, Kyo Young Song, Han Hong Lee, Min-Gew Choi, Ji Yeong An, Sang-Il Lee, Sang Hyub Lee, Do Joong Park

**Affiliations:** a Department of Internal Medicine, Seoul Metropolitan Government Boramae Medical Center, Seoul National University College of Medicine, Seoul; b Department of Surgery, Seoul National University Bundang Hospital, Seongnam-si; c Department of Surgery, Asan Medical Center, University of Ulsan College of Medicine, Seoul; d Department of Surgery and Research Institute for Convergence of Biomedical Science and Technology, Pusan National University Yangsan Hospital, Pusan National University School of Medicine, Yangsan; e Department of Surgery, Chosun University Hospital, Kwangju; f Department of Surgery, Kyoungpook National University Chilgok Hospital, Daegu; g Department of Surgery, Ajou University Hospital, Suwon; h Center for Gastric Cancer, National Cancer Center, Goyang; i Department of Surgery, Boramae Medical Center, Seoul; j Department of Surgery, Yonsei University Severance Hospital, Seoul; k Department of Surgery, Catholic University Seoul St. Mary’s Hospital, Seoul; l Department of Surgery, Samsung Medical Center, Sungkyunkwan University School of Medicine, Seoul; m Department of Surgery, Chungnam National University Hospital, Daejeon; n Department of Internal Medicine and Liver Research Institute, Seoul National University Hospital, Seoul National University College of Medicine, Seoul; o Department of Surgery and Cancer Research Institute, Seoul National University Bundang Hospital, Seoul National University Hospital, Seoul National University College of Medicine, Seoul, Republic of Korea

**Keywords:** bile reflux, gastrectomy, stomach neoplasms, ursodeoxycholic acid

## Abstract

**Background:**

Few studies have been conducted on the prevention of bile reflux in gastric cancer patients who have undergone gastrectomy. The aim of this study was to evaluate the efficacy and safety of ursodeoxycholic acid (UDCA) in preventing bile reflux after gastrectomy in patients with gastric cancer.

**Methods:**

This study was a secondary analysis of the PEGASUS-D trial, a randomized, double-blind, placebo-controlled clinical trial. Adults with a diagnosis of gastric cancer who underwent gastrectomy were enrolled. Eligible participants were randomly assigned to receive 300 mg of UDCA, 600 mg of UDCA, or placebo at a ratio of 1:1:1. UDCA and placebo were administered daily for 52 weeks. The primary outcomes included bile reflux symptoms at each time point, the percentage of participants with bile reflux, and the grade of gastritis.

**Results:**

Among 521 participants who underwent randomization, 151, 164, and 150 participants were analyzed from the 300 mg UDCA, 600 mg UDCA, and placebo groups, respectively. The difference in symptoms between the three groups was not significant. Bile reflux was less evident in the UDCA group than in the placebo group; however, this difference was significant only in the 300 mg group at 12 months postoperation (odds ratio, 0.44; *P*=0.0076). A significant reduction in gastritis was also observed in the 300 mg group at 12 months postoperation (odds ratio, 0.50; *P*=0.0368) compared to the placebo group.

**Conclusions:**

This study showed that UDCA administration significantly reduced bile reflux and gastritis by ~50% at the 12 months-postoperative follow-up in patients who underwent gastrectomy for gastric cancer.

## Introduction

Highlights

- Bile reflux gastritis and associated malignancies may arise as significant complications after gastrectomy.
- There has been no established treatment for bile reflux symptoms.
- The use of 300 mg of ursodeoxycholic acid, compared with placebo, resulted in a significantly decreased proportion of patients developing bile reflux and gastritis within 12 months after gastrectomy.

The incidence of gastric cancer (GC) has witnessed a decline over the past several decades^1,2^. Nonetheless, GC remains a major health problem, particularly in East Asian countries, including South Korea^2^. Presently, surgical resection is the sole curative option available for patients with localized and resectable GC^3^. In these patients, various reconstruction methods are employed following distal gastrectomy, such as Billroth I (B-I; gastroduodenostomy), Billroth II (B-II; gastrojejunostomy), and Roux-en-Y gastrojejunostomy (R-Y GJ).

Bile or duodenogastric reflux may be implicated in gastric and esophageal carcinogenesis^4–6^. Furthermore, histological evidence of bile reflux into the stomach is associated with cardia intestinal metaplasia, a likely precursor of cardia cancer^7^. More specifically, hydrophobic bile acids such as chenodeoxycholic acid, certain bile acid receptors, and gastric mucosal polyamine potentially play a role in GC initiation^8–11^. Although a previous meta-analysis reported that R-Y reconstruction was significantly superior to B-I and B-II reconstructions in terms of bile reflux frequency^12^, the latter have been preferred in Asia for their simplicity. Therefore, bile reflux gastritis and associated malignancies may arise as significant complications after gastrectomy, especially in Asian countries. Compared with acid reflux alone, the presence of bile in an acidic esophageal environment may be associated with more severe heartburn^13^. However, to date, there has been no established treatment for bile reflux symptoms^14^.

A previous clinical trial from the mid-1980s suggested that increasing the proportion of ursodeoxycholic acid (UDCA) in refluxed gastric bile reduces the pain and frequency of symptoms associated with bile reflux^15^. However, this finding may be limited in its applicability in the face of current advancements, and due to the trial’s 1-month treatment duration and sample size of only 12 patients. Recently, the PEGASUS-D study (efficacy and safety of UDCA for the prevention of gallstone formation after gastrectomy in patients with GC; a multicenter, randomized, double-blind, placebo-controlled study) revealed that administration of UDCA for 12 months significantly reduced the incidence of gallstones after gastrectomy for GC^16^. During the planning phase of the PEGASUS-D study, we hypothesized that UDCA would not only prevent gallstone formation but also reduce symptoms of bile reflux and its associated gastritis, based on prior findings. Therefore, bile reflux and related symptoms were also assessed throughout the trial. The present study aimed to evaluate the symptoms and endoscopic findings of bile reflux examined in the PEGASUS-D study and assess the efficacy of UDCA for treating bile reflux after gastrectomy.

## Methods

### Study design and participants

The PEGASUS-D trial is a multicenter, randomized, double-blind, placebo-controlled trial conducted in South Korea. A detailed methodological description of this trial has been presented previously^16^. The study protocol was approved by the institutional review boards of the 12 participating domestic institutions, and the trial was conducted in accordance with the principles of the Declaration of Helsinki.

Those who underwent total, distal, or proximal gastrectomy for GC were eligible to participate in the trial. Participants were enrolled between 26 May 2015 and 9 January 2017. The major inclusion criteria were D1+ or D2 lymph node dissection and an Eastern Cooperative Oncology Group performance status of 1 or lower. Additionally, the hepatic branch of the vagus nerve was routinely removed during lymph node dissection, while patients with pathological stage II or higher received adjuvant chemotherapy. The major exclusion criteria were active infection or inflammation, liver dysfunction, pre-existing gallstones, previous cholecystectomy, BMI >37 kg/m^2^, and hypersensitivity to UDCA.

### Randomization and interventions

Eligible participants who fulfilled the inclusion and exclusion criteria were randomly assigned to receive either 300 mg UDCA, 600 mg UDCA, or placebo at a ratio of 1:1:1 within 2 weeks postgastrectomy. An independent statistician generated a random allocation sequence using a stratified block randomization method, with institution, lymph node dissection range, and gastrectomy type as the stratification factors. Participants were assigned to the groups via a web response system at each site. Both the investigators and participants were blinded to the assignments.

All participants underwent abdominal ultrasonography to identify pre-existing gallstones at screening. Following screening, participants were administered UDCA 300 mg, UDCA 600 mg, or placebo for 52 weeks based on the group assignment via identical-shaped capsules (ursa-D capsule 300 mg, Daewoong Pharmaceutical Co., Ltd.) twice daily. Abdominal ultrasonography was performed every three months for 12 months to confirm gallstone formation.

### Assessment of bile reflux symptom and endoscopic evaluation

**Figure 1:**
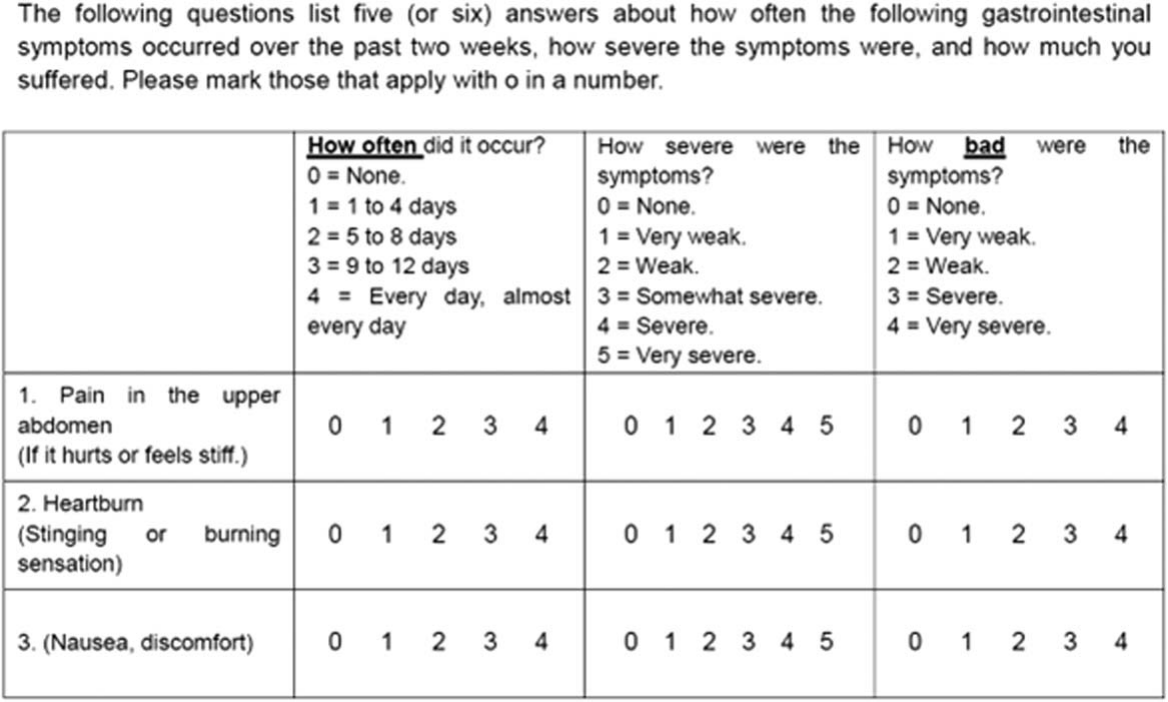
Questionnaire for evaluation of bile reflux symptoms.

Bile reflux symptoms were assessed using a self-administered questionnaire (Fig. 1) at every visit (baseline, and 3, 6, 9, and 12 months postoperation; maximum five times) or at the time of early termination or withdrawal to determine gallstone formation. The questionnaire was created by modifying the pre-existing questionnaires GerdQ^17^ and GERD-HRQoL^18^, which are used in the assessment of gastroesophageal reflux disease since there were no standard questionnaire forms for bile reflux. The participants completed the questionnaire in three areas: frequency, strength, and level of pain as related to the representative symptoms of bile reflux: upper abdominal pain, heartburn, and nausea.

Independent investigators performed upper gastrointestinal endoscopy at 3-months and 12-months postoperation (or at termination) to confirm actual bile reflux and recorded their findings. The presence of bile reflux was evaluated by observing the remnant stomach or the anastomotic area; bile presence was confirmed if a yellow liquid was observed in the assessed area^19^. The grade of gastritis was assessed according to the following criteria^19,20^: Grade 0, no flare; Grade 1, mild redness; Grade 2, a comb-shaped redness; Grade 3, severe redness and edema in the mucous membrane of the remnant stomach or throughout the anastomotic area. All participants who underwent total gastrectomy were recorded as Grade 0.

### Outcomes

The original primary outcome of the PEGASUS-D trial was the proportion of patients who developed gallstones within 12 months of gastrectomy. In the present secondary analysis, the primary outcomes included bile reflux symptoms at each time point, percentage of participants with bile reflux, and grade of gastritis as assessed by endoscopy.

### Statistical analysis

The original PEGASUS-D study was designed to demonstrate that UDCA administration was superior to placebo for gallstone prevention. Assuming that the proportion of patients with gallstone formation within 12 months postoperation was 7% in the UDCA groups^21^ and 18% in the placebo group^22,23^, the minimum sample size to detect a difference with 80% power (α=0.05, two-sided test) was 138 participants per group.

Outcomes were evaluated using the full analysis set (FAS), which comprised participants who underwent at least one evaluation of gallstone formation after randomization and did not violate the inclusion or exclusion criteria. Bile reflux symptom scores were compared between each UDCA group and the control group using the Analysis of Covariance (ANCOVA) model with a range of lymph node dissections and type of gastrectomy as covariates. The presence of bile reflux and gastritis was compared using a logistic regression model with a range of lymph node dissections and type of gastrectomy as covariates. The data were collected and validated using the Rave Electronic Data Capture System (Medidata Institute). All statistical analyses were performed using SAS version 9.4 (SAS Institute Inc., Cary, NC, USA).

## Results

### Study participants

**Figure 2:**
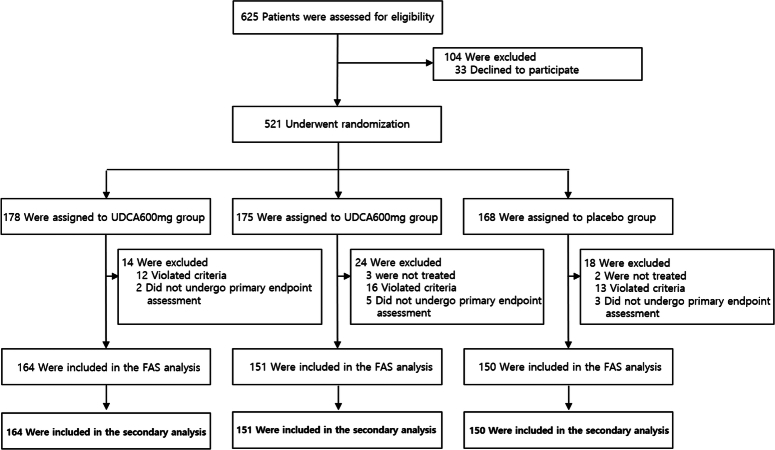
Patients flow diagram.

A total of 521 participants were randomly selected from 625 eligible patients across 12 participating institutions. Among them, 175, 178, and 168 participants were assigned to the 300 mg UDCA, 600 mg UDCA, and placebo groups, respectively. After follow-up, 164 in the 600 mg UDCA group, 151 in the 300 mg UDCA group, and 150 participants in the placebo group were included in the FAS analysis. These participants were included in this secondary analysis (Fig. 2). Most participants demonstrated over 80% adherence to the assigned medications: 151 out of 164 in the 600 mg group, 143 out of 151 in the 300 mg group, and 133 out of 150 in the placebo group. The three groups did not show any significant differences in terms of age, sex, BMI, smoking status, alcohol consumption, cancer stage, and other surgical factors. Detailed baseline characteristics were presented in a previous report^16^.

### Bile reflux

The bile reflux symptom scores of the participants at 3, 6, 9, and 12 months postoperation are presented in Table 1. Over time, the scores for each symptom (epigastralgia, heartburn, and nausea) and the overall symptom score decreased; however, the difference between the UDCA groups and control group was not significant. Table 2 shows the proportion of participants with bile reflux in each group at 3 and 12 months postoperation, as assessed by endoscopy. Bile reflux was less evident in the UDCA groups than in the placebo group; however, this difference was significant only in the UDCA 300 mg group at 12 months postoperation (odds ratio, 0.44; *P*=0.0076).

**Table 1:** Bile reflux symptoms at month 3, 6, 9, and 12.

Symptom	Time	UDCA 600 mg (*N*=164) (*P* ^a^)	UDCA 300 mg (*N*=151) (*P* ^a^)	Placebo (*N*=150)
Epigastralgia	Baseline^b^	1.75±2.92	2.14±3.08	1.92±3.07
	Month 3	1.10±2.56 (0.1163)	0.79±1.89 (0.7453)	0.78±1.77
	Month 6	0.95±2.29 (0.2327)	0.52±1.53 (0.4568)	0.69±1.82
	Month 9	0.94±2.21 (0.1748)	0.60±1.77 (0.3541)	0.52±1.61
	Month 12	0.52±1.83 (0.7401)	0.57±1.66 (0.2963)	0.49±1.53
Heartburn	Baseline^b^	1.57±1.79	0.70±2.06	0.27±1.09
	Month 3	0.57±1.90 (0.6983)	0.34±1.28 (0.0366)	0.36±1.30
	Month 6	0.47±1.83 (0.8273)	0.27±1.13 (0.2985)	0.25±1.11
	Month 9	0.23±1.03 (0.1331)	0.21±0.85 (0.0944)	0.15±0.86
	Month 12	0.33±1.19 (0.2589)	0.31±1.12 (0.1617)	0.18±0.87
Nausea	Baseline^b^	1.09±2.57	0.71±1.88	0.70±1.83
	Month 3	1.15±2.45 (0.3321)	1.35±2.48 (0.5308)	1.13±2.56
	Month 6	1.17±2.58 (0.6778)	0.99±2.24 (0.3638)	0.72±1.81
	Month 9	0.75±2.05 (0.6438)	0.51±1.33 (0.9441)	0.54±1.64
	Month 12	0.59±1.84 (0.1848)	0.42±1.46 (0.5389)	0.69±1.92
Total score	Baseline^b^	3.41±5.40	3.55±4.73	2.89±4.34
	Month 3	2.82±5.33 (0.8062)	2.47±4.04 (0.5344)	2.27±4.18
	Month 6	2.59±5.61 (0.3976)	1.79±3.26 (0.7066)	1.66±3.02
	Month 9	1.93±4.17 (0.8896)	1.32±2.67 (0.2370)	1.22±2.55
	Month 12	1.45±3.32 (0.4231)	1.31±3.03 (0.1428)	1.36±3.08

^a^ Testing for difference between each UDCA dose and placebo (ANCOVA model).

^b^ Insignificant *P*-value for difference among the treatment groups (ANOVA or Kruskal–Wallis test).

**Table 2:** Bile reflux at month 3 and 12 assessed by endoscopy.

Time	UDCA 600 mg (*N*=164)	UDCA 300 mg (*N*=151)	Placebo (*N*=150)
Month 3			
*N* (%)	31 (18.90)	30 (19.87)	41 (27.33)
Odds ratio (95% CI)	0.62 (0.36–1.08)	0.67 (0.38–1.16)	
*P*-value^a^	0.0902	0.1534	
Month 12			
*N* (%)	41 (25.00)	23 (15.23)	40 (26.67)
Odds ratio (95% CI)	0.76 (0.44–1.32)	0.44 (0.24–0.80)	
*P*-value^a^	0.3273	0.0076	

a Testing for difference between each UDCA dose and placebo (logistic regression model).

The proportion of participants with bile reflux gastritis (grades 1–3) at 3 and 12 months is presented in Table 3. Again, a significant reduction in gastritis was observed only in the 300 mg UDCA group at 12 months postoperation (odds ratio, 0.50; *P*=0.0368). The proportion of bile reflux and gastritis was increased in patients who underwent B-II anastomosis in the subgroup analysis (Table 4). The outcomes for B-I and R-Y are presented in the supplementary tables (Tables S1 and S2, Supplemental Digital Content 1, http://links.lww.com/JS9/D511, respectively). UDCA 600 mg was superior to UDCA 300 mg in preventing bile reflux, particularly in patients with B-II anastomosis assessed at 3 months postoperation. However, there was no significant difference in bile reflux between the groups in patients with B-I and R-Y anastomoses. Finally, no significant association was observed between bile reflux and gallstone formation within 12 months postoperation.

**Table 3:** Gastritis at month 3 and 12 assessed by endoscopy.

Time	UDCA 600 mg (*N*=164)	UDCA 300 mg (*N*=151)	Placebo (*N*=150)
Grades 1–3			
Month 3			
*N* (%)	26 (15.85)	27 (17.88)	36 (24.00)
Odds ratio (95% CI)	0.59 (0.33–1.06)	0.69 (0.39–1.23)	
*P*-value^a^	0.0737	0.2107	
Month 12			
*N* (%)	29 (17.68)	18 (11.92)	29 (19.33)
Odds ratio (95% CI)	0.78 (0.43–1.42)	0.50 (0.26–0.96)	
*P*-value^a^	0.4215	0.0368	
Grades 2–3			
Month 3			
*N* (%)	9 (5.49)	8 (5.30)	9 (6.00)
Odds ratio (95% CI)	0.93 (0.36–2.43)	0.90 (0.34–2.42)	
*P*-value^a^	0.8708	0.8327	
Month 12			
*N* (%)	7 (4.27)	3 (1.99)	4 (2.67)
Odds ratio (95% CI)	1.52 (0.43–5.34)	0.69 (0.15–3.16)	
*P*-value^a^	0.5078	0.6297	

^a^ Testing for difference between each UDCA dose and placebo (logistic regression model).

**Table 4:** Bile reflux and gastritis (grade 1-3) at month 3 and 12 assessed by endoscopy in subjects with Billroth II

Time	UDCA 600 mg (*N*=47)	UDCA 300 mg (*N*=32)	Placebo (*N*=43)
Month 3			
Bile reflux			
*N* (%)	13 (27.66)	11 (34.38)	25 (58.14)
Odds ratio (95% CI)	0.27 (0.11–0.67)	0.38 (0.14–1.04)	
*P*-value^a^	0.0051	0.0599	
Gastritis (grades 1–3)			
*N* (%)	12 (25.53)	11 (34.38)	24 (55.81)
Odds ratio (95% CI)	0.27 (0.11–0.67)	0.42 (0.16–1.08)	
*P*-value^a^	0.0049	0.0719	
Gastritis (grades 2-3)			
*N* (%)	3 (6.38)	3 (9.38)	5 (11.63)
Odds ratio (95% CI)	0.53 (0.12–2.38)	0.81 (0.18–3.74)	
*P*-value^a^	0.4054	0.8030	
Month 12			
Bile reflux			
*N* (%)	14 (29.79)	12 (37.50)	19 (44.19)
Odds ratio (95% CI)	0.39 (0.14–1.05)	0.59 (0.20–1.73)	
*P*-value^a^	0.0660	0.3347	
Gastritis (grades 1–3)			
*N* (%)	14 (29.79)	12 (37.50)	16 (37.21)
Odds ratio (95% CI)	0.60 (0.23–1.58)	0.890 (0.29–2.24)	
*P*-value^a^	0.3138	0.6705	
Gastritis (grades 2–3)			
*N* (%)	2 (4.26)	1 (3.13)	2 (4.65)
Odds ratio (95% CI)	0.84 (0.11–6.35)	0.58 (0.05–6.82)	
*P*-value^a^	0.8743	0.6757	

^a^ Testing for difference between each UDCA dose and placebo (logistic regression model).

### Adverse events

As outlined in the previous report, adverse events were rare across all participants^16^. The proportions of participants experiencing any adverse events were 1.7% in the 600 mg group, 4.7% in the 300 mg group, and 1.8% in the placebo group. The most common symptom was nausea, occurring in 4 of 516 participants (0.8%), followed by skin rash in 3 of 516 participants (0.6%). There were no reports of serious adverse events or deaths related to the treatment.

## Discussion

In this secondary analysis of the PEGASUS-D trial, UDCA demonstrated potential efficacy in mitigating bile reflux and associated gastritis in patients who had underwent gastrectomy for GC. In addition to its preventive role in gallstone formation, as shown by the PEGASUS-D trial, an additional role for UDCA in bile reflux reduction is suggested by the present study. These findings present compelling evidence for the prospective therapeutic utility of UDCA for bile reflux in patients undergoing gastrectomy, an area that has till date lacked established interventions. Furthermore, the findings provide clinical evidence of the versatility of UDCA, a very safe drug. It is crucial to note, however, that this study did not definitively confirm the impact of UDCA on alleviating bile reflux symptoms.

Bile reflux into the esophagus via the stomach has been recognized as a significant factor in the pathophysiology of gastroesophageal reflux disease, especially in patients who exhibited a poor response to proton pump inhibitor treatment. The predominant symptoms of bile reflux are heartburn and chest or epigastric pain; notably, there are no bile reflux-specific symptoms^24^. The presence of bile reflux has been associated with gastric and esophageal carcinogenesis, as mentioned above. Consequently, it is crucial to minimize bile reflux to the greatest extent feasible in patients undergoing gastrectomy.

The choice of reconstruction method has had a notable impact on the presence and degree of bile reflux in patients who had underwent distal gastrectomy. A recent network meta-analysis revealed that R-Y reconstruction showed significant superiority over B-I and B-II reconstruction in terms of remnant gastritis, with ORs of 0.33 and 0.40, respectively^12^. Various reconstruction methods, including Braun^25^, uncut R-Y^26^, and double tract reconstruction^27^, have been introduced to diminish bile reflux and improve postoperative quality of life. In the present study, the proportion of participants with bile reflux was the highest among those who underwent B-II anastomosis, at 58.14% at postoperative month 3 and 44.19% at postoperative month 12 in the placebo group. Additionally, our findings revealed that daily UDCA 300 mg administration after gastrectomy significantly reduced bile reflux with an OR of 0.44 (*P*=0.0076) assessed at postoperative month 12, when compared with placebo. Similarly, daily administration of 300 mg UDCA significantly diminished remnant gastritis (grades 1–3) at postoperative month 12, with an OR of 0.50 (*P*=0.0368), when compared with placebo. In our subgroup analysis, this UDCA effect was more pronounced in patients who underwent B-II reconstruction (especially 3 months after surgery), but not in patients who underwent B-I or R-Y reconstruction (Supplementary Tables, Supplemental Digital Content 1, http://links.lww.com/JS9/D511). However, UDCA administration did not improve bile reflux symptoms, which were evaluated every 3 months.

UDCA therapy has been found to significantly increase its proportion in gastric bile, accounting for ~50% of the total bile acids^15^. Therefore, it can diminish bile-induced inflammation by decreasing the proportion of toxic bile acids^28^, and increase the hydrophilicity index of the circulating bile acid pool^29^. Ozkaya *et al*.^30^ reported that UDCA treatment with a duration of 6 months decreased the degree of epidermal growth factor (EGF) positivity in the gastric mucosa, indicating mucosal healing. Furthermore, UDCA may reduce bile reflux itself, although the exact mechanism remains unknown^15,30^. It is postulated that UDCA exerts this potential effect by suppressing small intestinal inflammation and increasing mucin production^31^. However, ultimately further studies are required to elucidate the mechanism of reducing bile reflux by UDCA administration to gain a deeper understanding of the therapeutic potential of UDCA in managing bile reflux-related complications.

In the present study, a significant protective effect against bile reflux and associated gastritis was observed only in in the 300 mg UDCA group. However, as this study was not designed to compare 300 and 600 mg UDCA, it would be premature to conclude that 300 mg is superior to 600 mg based on our findings. Moreover, 600 mg UDCA showed significantly positive outcomes in patients with B-II anastomosis, although these results did not persist until the 12 month-follow-up. Although the exact mechanism is unclear, higher doses of UDCA may be more beneficial for patients with increased bile reflux. It is thought that a high-dose of UDCA is helpful initially but then becomes less effective as the body goes through a physiologic adjustment period. Additionally, the unabsorbed portion of higher UDCA doses may reach the colon, where it could be converted into hepatotoxic bile acids. In particular, as previous studies have shown, high-dose UDCA can be harmful to patients with primary sclerosing cholangitis^32,33^; thus, the UDCA dose should be determined based on the individual situation, taking into account factors such as their specific condition and medical history.

In addition to the previous UDCA trial on bile reflux described in the introduction^15^, a more recent clinical trial was conducted in Iran^34^. The authors concluded that adding UDCA to sucralfate and omeprazole was not clinically effective in patients with bile reflux gastritis (*n*=60). However, they only examined bile reflux symptoms (pain, heartburn, and bloating) and did not provide information on the UDCA dosage used. Furthermore, it is difficult to generalize the results of the aforementioned trials due to their limited reporting and the absence of baseline characteristics of the enrolled participants. Contrastingly, the Iranian study reported that after three weeks, there was a significant improvement in symptoms in both the treatment and control groups. This suggests that proton pump inhibitor therapy with sucralfate is beneficial in treating bile reflux symptoms.

Aforementioned study on the effect of UDCA on bile reflux gastritis reported a decrease in EGF positivity following UDCA treatment^30^. This study included only 31 postcholecystectomy patients and compared EGF staining results before and after six months of UDCA therapy. However, unlike our study, the authors did not account for any symptoms potentially related to bile reflux. Nevertheless, they proposed mechanisms for the effect of UDCA on bile reflux, noting that EGF plays an important role in maintaining mucosal integrity^35^. This finding is further supported by an *in vitro* study demonstrating that UDCA protects against intestinal barrier breakdown through EGF receptor- and cyclooxygenase-2 (COX-2)-dependent mechanisms^36^. The study showed a dose-dependent increase in COX-2 expression with UDCA, with the greatest increase observed at 200 µM rather than 400 µM. This may be related to our study’s findings, which showed significant results with 300 mg of UDCA, but not with 600 mg.

In terms of safety, UDCA was associated with very few adverse events in all groups during the study, with no more adverse events in the 600 mg group. These results suggest that UDCA 300 mg or 600 mg for 1 year is very safe for GC surgery patients, and are consistent with previous reports^37^. The long-term safety of UDCA has been extensively studied, particularly in patients with primary biliary cholangitis, where it remains the standard of care^38^.

The present study, although not primarily designed to assess the outcomes of bile reflux, provided valuable insights on bile reflux treatment, given that there have been only a few pharmacological treatment studies assessing this issue. However, this study has several limitations. First, due to the study’s original design, a direct statistical comparison between the UDCA and control groups in terms of symptoms, presence of reflux, and severity of gastritis was challenging to establish. Second, the bile reflux symptom questionnaire devised for this study was not validated, as is no standardized questionnaire for bile reflux symptoms. Third, the endoscopic evaluation was performed by independent investigators at each participating institution. This approach introduces the possibility of variability in the assessment of bile reflux, potentially influencing our results. Furthermore, endoscopic evaluations were not performed in some participants since endoscopic evaluation was not a criterion for inclusion in the FAS.

In conclusion, our study demonstrated that UDCA administration significantly reduced bile reflux and gastritis evaluated at 12 months postoperatively by ~50% in patients who underwent gastrectomy for GC. These results were more pronounced with a lower dose of UDCA (300 mg), whereas a higher dose (600 mg) may be more beneficial in patients with B-II anastomosis in the early postoperative phase. In the future, large-scale studies to effectively compare the different dosages and evaluate the effectiveness of UDCA in patients using the same surgical anastomosis method are warranted. In particular, there is an urgent need to plan large prospective studies to determine appropriate UDCA doses, especially for patients with B-II anastomosis, where bile reflux is common.

## Ethical approval

The study protocol was approved by the Seoul National University Hospital Institutional Review Boards (IRB No. 2104-198-1216).

## Consent

All participants provided written informed consent before enrollment.

## Source of funding

This study was supported by a research grant from Daewoong Pharmaceutical Co Ltd.

## Author contribution

Dr D.J.P.: had full access to all of the data in the study and takes responsibility for the integrity of the data and the accuracy of the data analysis; D.K.J., Y.S.P., M.-W.Y., S.-Y.R., S.H.L., and D.J.P.: concept and design; D.K.J., M.-W.Y., S.-H.H., S.-Y.R., O.K.K., H.H., H.M.Y., B.W.E., H.S.A., T.S., K.Y.S., H.H.L., M.-G.C., J.Y.A., S.-I.L., S.H.L., and D.J.P.: acquisition, analysis, or interpretation of data; D.K.J., Y.S.P., S.H.L., and D.J.P.: drafting of the manuscript; H.S.A., S.H.L., and D.J.P.: statistical analysis; S.H.L. and D.J.P.: obtained funding; D.K.J., Y.S.P., M.-W.Y., S.-Y.R., B.W.E., H.S.A., J.Y.A., S.H.L., and D.J.P.: administrative, technical, or material support; S.-Y.R., K.Y.S., J.Y.A., S.H.L., and D.J.P.: supervision. All authors contributed in critical revision of the manuscript for important intellectual content.

## Conflicts of interest disclosure

Dr S.H. Lee reported receiving speaker honoraria and grants from Daewoong Pharmaceutical Co Ltd during the conduct of the study. Dr Yoo reported receiving grants from Daewoong Pharmaceutical Co Ltd during the conduct of the study. Dr Y. S. Park reported receiving grants from Daewoong Pharmaceutical Co Ltd during the conduct of the study. Dr D. J. Park reported receiving speaker honoraria from Daewoong Pharmaceutical Co Ltd during the conduct of the study; and a research grant from Medtronic outside the submitted work. No other disclosures were reported.

## Research registration unique identifying number (UIN)

ClinicalTrials.gov Identifier: NCT02490111.

## Guarantor

Do Joong Park, the corresponding author.

## Data availability statement

Any datasets during the current study are available upon reasonable request.

## Provenance and peer review

Our paper was not invited and we agree with your policy.

## Supplementary Material

SUPPLEMENTARY MATERIAL
