# Supplementary material for: Efficacy of ursodeoxycholic acid for bile reflux after distal gastrectomy in patients with gastric cancer: a secondary analysis of the PEGASUS-D randomized clinical trial
Source: Int J Surg. 2024 Oct 18;110(12):7824–31. doi: 10.1097/JS9.0000000000002127 (PMC11634197; doi:10.1097/JS9.0000000000002127)
Supplement: SUPPLEMENTARY MATERIAL [file js9-110-7824-s001.pdf]

Supplementary tables

Table S1. Bile reflux at month 3 and 12 by endoscopy in subjects with Billroth I

Time		UDCA 600mg (N=34)	UDCA 300mg (N=30)	Placebo (N=26)
Month 3	N (%)	12 (35.29)	9 (30.00)	7 (26.92)
	Odds ratio (95% CI)	1.36 (0.44-4.23)	1.23 (0.37-4.06)	
	P value*	0.6071	0.7449	
Month 12	N (%)	18 (52.94)	9 (30.00)	11 (42.31)
	Odds ratio (95% CI)	1.30 (0.43-3.94)	0.49 (0.15-1.58)	
	P value*	0.6711	0.2359	

* Testing for difference between each UDCA dose and placebo (logistic regression model)

Table S2. Bile reflux at month 3 and 12 by endoscopy in subjects with Roux-en-Y

Time		UDCA 600mg (N=52)	UDCA 300mg (N=58)	Placebo (N=53)
Month 3	N (%)	2 (3.85)	4 (6.90)	3 (5.66)
	Odds ratio (95% CI)	0.68 (0.11-4.28)	1.22 (0.26-5.75)	
	P value*	0.6621	0.7978	
Month 12	N (%)	4 (7.69)	1 (1.72)	2 (3.77)
	Odds ratio (95% CI)	2.02 (0.33-12.21)	0.43 (0.04-5.05)	
	P value*	0.4399	0.4784	

* Testing for difference between each UDCA dose and placebo (logistic regression model)
